# Oral microbial profiles of individuals with different levels of sugar intake

**DOI:** 10.1080/20002297.2017.1355207

**Published:** 2017-08-01

**Authors:** Mette K. Keller, Christine A. Kressirer, Daniel Belstrøm, Svante Twetman, Anne C. R. Tanner

**Affiliations:** ^a^ Department of Odontology, Faculty of Health and Medical Sciences, University of Copenhagen, Copenhagen, Denmark; ^b^ Department of Microbiology, The Forsyth Institute, Cambridge, USA; ^c^ Department of Oral Medicine, Infection and Immunity, Harvard School of Dental Medicine, Harvard University, Boston, USA

**Keywords:** Caries, sugar, oral microbiota, HOMI*NGS*

## Abstract

The aim was to compare the oral microbial profiles in young adults with an intake of free sugars above or below the current recommendations by the WHO for sugar consumption. Seventy subjects completed a Quantitative Food Frequency Questionnaire to establish the proportion of free sugars in relation to the total energy intake (% E). Subjects with <5% E (*n* = 30) formed the low-sugar group, while those with ≥5% E (*n* = 40) were regarded as reference group. Saliva and plaque samples were analyzed by qPCR, and 52 of the plaque samples were assayed by HOMI*NGS*. The HOMI*NGS* analysis revealed a comparable core microbiota in plaque samples with *Streptococcus*, *Leptotrichia*, *Actinobaculum*, and *Veillonella* as predominant. No major differences between groups were revealed by α-diversity testing (*p *= 0.83), principal component analysis, or correspondence analysis. Higher relative abundance of *Streptococcus sobrinus* and *Prevotella melaninogenica* was observed in plaque samples in the reference group. By qPCR, *Scardovia wiggsiae* was associated with elevated sugar intake. The findings suggests that the amount of ingested sugars had a marginal influence on microbial profiles in dental plaque and saliva. However, some caries-associated species were less abundant in the dental plaque of the low sugar group.

## Background

The Human Microbiome Project has provided insight into microbial ecology, suggesting that bacterial biofilms have co-evolved with humans and play an important role in health and well-being. Contemporary molecular biological techniques, including next-generation sequencing (NGS) methods, have improved our understanding of complex biofilm compositions and their functions [1]. For the oral cavity, the Human Oral Microbiome Database contains >700 taxa, including named (54%) and unnamed (14%), and cultivable and uncultivated (32%) phylotypes (www.homd.org/). The composition of the oral microbiota is thought to play an active role in the maintenance of oral health [2]. Dental caries, the world’s most common oral disease, is a biofilm-mediated condition resulting from interactions between the commensal microbiota, host susceptibility, and environmental factors, particularly the diet [3].

Fermentable carbohydrates, especially sugars, are major factors that drive the caries-promoting dysbiosis in the oral microbiota, and the relationship between the amount and frequency of intake of refined sugar and clinical caries development has been well established [4,5]. Recently, it has been suggested that limiting the intake of free sugars to either <10% or even <5% of the total energy intake would lower the risk of dental caries [5]. Altered salivary microbial profiles in adults with untreated caries compared to control subjects has been been shown previously [6], but less is known about the impact of sugar consumption on the composition and function of the microbiota in saliva and dental plaque. The aim of this study was therefore to compare the oral microbial profiles in young healthy subjects with a habitual intake of free sugars below and above the recent World Health Organization (WHO) guidelines of either <5% or <10% of the total energy consumption [7].

## Material and methods

### Study group

In total, 90 young adults of both sexes volunteered to participate after written and verbal information about the study. The majority of participants were female university students characterized by a healthy life-style; none were obese. The diet of the volunteers was screened by a rapid Food Frequency Questionnaire (FFQ) consisting of 28 items (see supplemental data). Inclusion criteria for further participation was having a varied diet with either more than five or less than two intakes per day containing refined sugars in food and beverages for at least 3 months. Exclusion criteria were (1) general and systemic diseases or any drug use that could affect normal salivary functions, (2) recent or frequent antibiotic treatment during the last year, and (3) abundant plaque reflective of inadequate oral hygiene. After screening, 70 participants were selected and completed a comprehensive FFQ comprising 180 food items [8,9]. The dietary data were used to establish the proportion of free sugars in relation to the total energy with the aid of a computer program (DANKOST) based on the Danish food composition from The Danish Diet Databank [10]. Subjects were thereafter divided into a low-sugar group with <5% sugar intake in the diet and a reference group with daily sugar intake ≥5% of the total energy.

The study was granted exemption from requiring ethics approval by the National Danish Ethics Committee (H-1-2013-079). The study was approved by the Danish Data Authorities (2013-41-2592).

### Clinical examination and sampling

The dentition of the study participants was examined by a single examiner at the Dental School of the University of Copenhagen. The prevalence of dental caries was scored according to the WHO criteria [11] and expressed as decayed, missed, and filled teeth (DMFT). No radiographs were taken. Information on current medication, tobacco use, oral hygiene habits (brushing teeth twice per day = 2, once per day = 1, less than once per day = 0), and use of fluoride was collected through a structured interview at the clinical examination. Paraffin-stimulated whole saliva was collected during a 5 min period at least 2 h after brushing teeth and any food intake. Supragingival plaque samples were collected with a sterile explorer and pooled from the buccal and interdental surfaces of the anterior and posterior teeth. All samples were immediately frozen and stored at −80°C until further analysis.

### *DNA extraction and quantitative* polymerase chain reaction

Bacterial DNA was extracted using a protocol modified from the Epicentre MasterPure DNA Purification Kit. In brief, the samples were suspended in 100 µL of TE buffer supplemented with 1 µL of Ready-lyse Lysozyme and incubated at 37°C overnight. Next, 100 µL of 2× T&C Lysis Solution and 1 µL of Proteinase K were added to the suspensions and incubated at 65°C for 30 min. Samples were placed on ice before 117 µL of MPC Protein Precipitation Reagent was added, and the samples were vortexed and centrifuged. The supernatant was transferred to 400 µL of isopropanol and centrifuged after 10 min. The pellet was then washed twice in 75% ethanol, dried, and resuspended in 25 µL of TE buffer. The DNA concentration varied between 18 and 4,800 ng/µL in 25 µL o fTE buffer. The samples were diluted to obtain concentrations of 10 ng/µL and 50 ng/µL for the plaque and saliva samples, respectively. Quantitative PCR (qPCR) was performed on the saliva and plaque samples. Briefly, the qPCR reaction mixtures contained 20 µL of total volume consisting of Roche SYBR Green master mix 2× (10 µL), 20 µM of primers (0.25 µL), PCR grade water (5.5 µL), and DNA (4 µL). The qPCR conditions were as previously described for *Scar-dovia wiggsiae* [12] using primers forward 5ʹ- GTGGACTTTATGAATAAGC-3ʹ and reverse 5ʹ-CTACCGTTA AGCAGTAAG-3ʹ; *Strepto-coccus mutans* [13] using primers forward 5ʹ-TCGCGAAAAAGATAAACAAACA-3ʹ and reverse 5ʹ-GCCCCTTCACAGTTGGTTAG-3ʹ; total *Lactobacillus* species [14] using primers forward 5ʹ-TGGAAACAGRTGCTAATACCG-3ʹ and reverse 5ʹ-GTCCATTGTGGAAGATTCCC-3ʹ; and total bacteria using primers forward 5′-TGGAGCATGTGGTTTAATTCGA-3′ and reverse 5′-TGCGGGACTTAACCCAACA-3′ [15,16].

### HOMI*NGS analysis*

The sequencing of 16S rDNA was analyzed according to Gomes et al. [17], as modified by Gregory et al. [18]. In brief, the Illumina-based NGS approach with bioinformatic ‘probes’ allows for identification of up to 600 oral taxa at species level. In the procedure, 50 ng of DNA was PCR amplified using V3V4 primers and 5 Prime Hot Master Mix. PCR amplicons were cleaned up using Ampure beads. Next, 100 ng of each library was pooled together, ran on a gel, gel-extracted, and ran on a bioanalyser for quantification. Then, 4 nM of the library was diluted down, and 12 pM of the library was spiked in 20% Phix (Illumina, San Diego, CA) and loaded on the Miseq (Illumina). The primers used were: Region V3V4: ~341F (forward primer) AATGATACGGCGACCACCGAGATCTACACTATGGTAATTGTCCTACGGGAGGCAGCAG and ~806R (reverse primer) CAAGCAGAAGACGGCATACGAGATTCCCTTGTCTCC AGTCAGTCAGCCGGACTACHVGGGTWTCTAAT. Species-specific, 16S rRNA-based oligonucleotide ‘probes’ were used in a BLAST program (ProbeSeq for HOMI*NGS*) to identify the frequency of oral bacterial targets. Five hundred and ninety-eight oligonucleotide probes of 17–40 bases targeted individual oral bacterial species or, in some cases, a few closely related species. An additional panel of 94 genus-specific probes was used. Outputs were expressed in Microsoft Excel spreadsheets as % frequencies of the target.

### Statistical methods

Data from qPCR were processed in IBM SPSS Statistics for Windows v21 (IBM Corp., Armonk, NY) using the Mann–Whitney *U*-test to compare the concentrations of bacteria in the two diet groups. Fisher’s exact test was performed to determine if there was an association between diet group and low (<1,000 pg DNA/µL) or high (≥1,000 pg DNA/µL) concentrations of *S. wiggsiae*. Data from HOMI*NGS* were processed by MeV v4.9 [19] and GraphPad Prism (GraphPad Software, Inc., La Jolla, CA) to calculate the Shannon diversity index (α-diversity). Data reduction and graphic presentation using principal component analysis and correspondence analysis were performed. Comparisons of the two diet groups at probe level were made using the Mann–Whitney *U*-test. An adjusted *p*-value of <0.05 was considered significant. Demographics of subjects with and without sufficient DNA in plaque samples were compared by Student’s *t*-test (age, sex, DMFT) and Mann–Whitney *U*-test (oral hygiene).

Sample size was based on results from a previous study analyzed with the same assays [20,21]. It was estimated that approximately 30 subjects in each sugar category would be sufficient in order to avoid type I and type II errors.

## Results

### Study groups

The characteristics of the participants in the two groups are shown in Table 1. The low-sugar group consisted of 30 subjects, with free sugars constituting from 1.6% to 4.9% of the total energy intake. The reference group had 40 participants, with an intake of free sugars between 5.0% and 21.0%. The groups were balanced with respect to age, sex, and oral hygiene, but the ≥5% group displayed a significantly higher caries experience (*p* < 0.05). Insufficient DNA was obtained from 18 plaque samples, which reduced the groups as shown in Table 2.

**Table 1.** Background data for the two groups with less or more added sugar than 5% of the total energy intake

	<5% of total energy consumption, *n* = 30	≥5% of total energy consumption, *n* = 40
Age, years (*SD*)	27 (8.8)	30 (7.8)
Sex, F/M (%)	24/6 (80/20)	31/9 (78/22)
DMFT (*SD*)	2.0 (3.6)	4.6 (4.5)
Oral hygiene 0/1/2	1/15/14	1/19/20
Sugar content of total energy (E %)	3.1 (1.6)	9.0 (3.7)

Oral hygiene: less than once per day = 0; once per day = 1; twice per day = 2.

*SD*, standard deviation.

**Table 2.** Comparison of the characteristics of subjects with samples included for assay with subjects with samples excluded

	Plaque samples included, *n* = 52		Plaque samples excluded, *n* = 18	
	<5% (*n* = 20)	≥5% (*n* = 32)	<5% (*n* = 10)	≥5% (*n* = 8)
Age, years (*SD*)	28 (10.2)	30 (7.8)	25 (1.8)	32 (8.0)
Sex, F/M (%)	16/4 (80/20)	25/7 (78/22)	8/2 (78/22)	6/2 (71/29)
DMFT (*SD*)	2.2 (3.9)	4.7 (4.7)	1.7 (2.7)	4.0 (3.8)
Oral hygiene 0/1/2	0/12/8	1/14/17	1/3/6	0/5/3

Added sugar either <5% of the total energy consumption or >5% of the total energy consumption.

### *HOMI*NGS analysis: sequencing

In the 52 plaque samples analyzed by HOMI*NGS*, positive reads for 550 probe sequences were found, which corresponded to 75% coverage of the 768 probes available in the database. Of the 550 probe-sequences, 439 were at species level and 111 at genus level. On average, a mean of 103,778 (range 11,050–162,714) sequences were generated per sample, out of which 72.5% (range 54.0–90.5%) and 50.2% (range: 30.7–85.6%) could be identified at genus and species level, respectively.

### *HOMI*NGS analysis: microbial profiles by sugar groups

**Figure 1.**
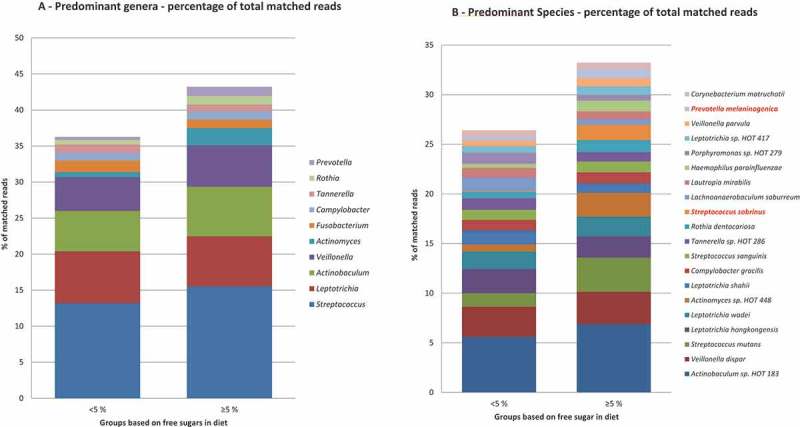
Relative abundance of predominant species- and genus-level probe targets. (a) Relative abundance of the 14 most predominant genus-level probe targets in each group. (b) Relative abundance of the 20 most predominant species-level probe targets in each group. An adjusted *p*-value of <0.05 was considered statistically significant and is highlighted in red.

**Figure 2.**
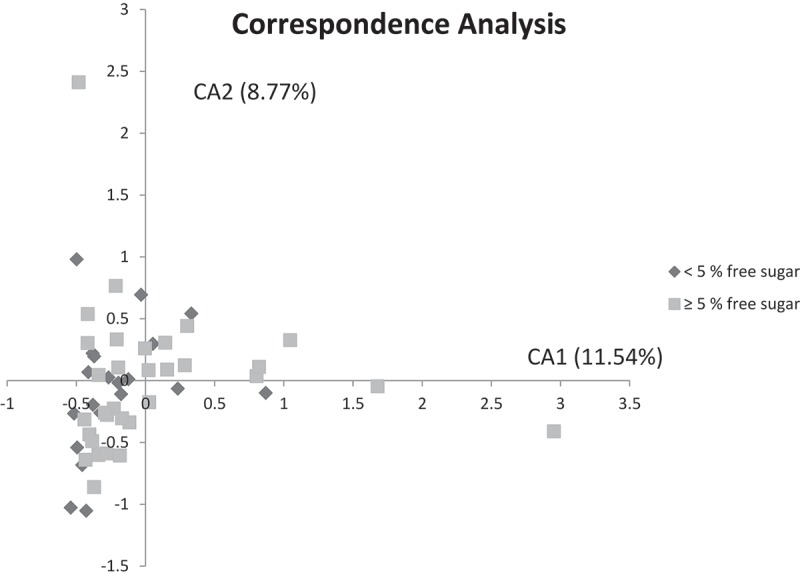
Correspondence analysis visualized two-dimensionally with axes expressed as the two foremost inertia values accounting for a cumulative inertia of 20.31%. Samples from the low-sugar group (dark gray) and samples from reference group samples (light gray).

The predominant bacterial genera detected in both sugar groups were *Streptococcus*, *Leptotrichia*, *Actinobaculum*, *Veillonella*, and *Actinomyces*, which constituted approximately 35% of all bacterial identifications (Figure 1a), and the relative abundance was comparable in both groups. Although not statistically significant, a threefold higher prevalence of *Actinomyces* was, however, noted in samples from the reference than from the low-sugar groups (2.4% vs. 0.7%). *Lactobacillus* was more abundant in the reference group than in the low-sugar group (0.052% vs. 0.009%). The predominant bacterial species are displayed in Figure 1b. *Actinobaculum* sp. HOT 183 was detected at 5.6% in the low-sugar group and at 6.9% in the reference group. *Streptococcus sobrinus* (1.5% vs. 0.1%) and *Prevotella melaninogenica* (0.9% vs. 0.3%) were significantly more prevalent in the reference group than in the low-sugar group (*p* < 0.05). Several species, including *S. mutans*, *S. wiggsiae* (0.33% vs. 0.69%), *Rothia dentocariosa*, *Veillonella parvula*, and *Actinomyces* sp. HOT 448 were also more prevalent in the reference group, but that was not statistically significant. There was comparable α-diversity in the plaque samples from the references (*M* = 2.70) and the low-sugar intake group (*M* = 2.72; *p *= 0.83). Furthermore, principal component analysis and the correspondence analysis of all species in the plaque samples did not separate the microbial profiles of the two groups (Figure 2 and supplemental data).

### qPCR data

The concentrations of DNA in the plaque and saliva samples are shown in Table 3. In the plaque samples, *S. wiggsiae* was detected at higher levels in the reference group (*p *< 0.05) when compared to the low-sugar group. Fisher’s exact test showed a statistically significant association between the diet group and the concentration of *S. wiggsiae* (*p* = 0.009). No other differences in taxa detected by qPCR were observed between the two sugar consumption groups.

**Table 3.** The concentration of DNA (pg DNA/µL) for *Scardovia wiggsiae, Streptococcus mutans*, total *Lactobacillus*, and total bacteria in plaque and saliva samples from subjects with different levels of sugar intake

	Plaque, *n* = 52			Saliva, *n* = 70		
	<5%	≥5%		<5%	≥5%	
	Median	Median	*p*	Median	Median	*p*
*Scardovia wiggsiae*	5.6 × 10^1^	2.6 × 10^3^	0.003	3.0 × 10^2^	4.0 × 10^2^	0.476
*Streptococcus mutans*	4.7 × 10^4^	3.9 × 10^4^	0.840	5.7 × 10^3^	2.9 × 10^3^	0.976
Total *Lactobacillus*	2.8 × 10^4^	3.0 × 10^4^	0.690	4.2 × 10^4^	3.1 × 10^4^	0.887
Total bacteria	1.2 × 10^6^	1.1 × 10^6^	0.601	1.1 × 10^6^	7.0 × 10^5^	0.691

## Discussion

In this study investigating the relation between contrasting sugar intake and microbial profiles with DNA-based technologies, interesting findings were observed in microbial profiles between sugar groups at the species but not the genus level. The participants of the low-sugar group were less frequently colonized by *S. sobrinus* and *P. melaninogenica* and displayed lower levels of a number of caries-associated bacteria such as *S. mutans*, *S. wiggsiae*, *Veillonella*, and *Actinomyces* species. *S. sobrinus* is frequently associated with dental caries [22] and has been described as both highly acidogenic and acid tolerant [23]. While Gross et al. [24] did not find *P. melaninogenica* or other *Prevotella* spp. to be associated with caries, they have been observed more frequently in children with early childhood caries [22] and were found to be associated with white-spot lesions in children with low levels of *S. mutans* [24]. A recent comparison of the genomes of several strains of *S. mutans* and *S. sobrinus* found *S. sobrinus* strains to contain more genes for possible aciduric and acidogenic properties than the *S. mutans* strains [25]. Clinical studies have linked *S. mutans* and *S. sobrinus* to both caries progression [25] and early childhood caries [22], and the concurrent appearance of *S. mutans* and *S. sobrinus* seem to be strongly associated with caries status [26,27]. Other bacteria that have been associated with carious dentine are *S. wiggsiae*, *R. dentocariosa*, *Actinomyces*, and *Veillonella* species [28]. Interestingly, *S. wiggsiae* was found to be in low abundance in the low-sugar group using the PCR assay. Similar data are available from preschool children with a cariogenic diet [29] and from studies comparing caries-susceptible with caries-free children [30]. Collectively, findings from both previous studies and the present study provide some indirect support for the ecological plaque hypothesis, suggesting that the sugar content of the diet may influence the selection of a cariogenic segment of the dental plaque microbiome.

In a previous study, it was not possible to unveil a significant impact of the general diet on the microbial profile in mixed saliva [31]. This study therefore focused on the consumption of refined sugars. The rationale to use the 5% threshold for free-sugar intake and the subsequent group assignment was that this level is considered safe for teeth, Moynihan and Kelly [5] stated that below this level, any person is highly unlikely to develop caries, even in the absence of daily fluoride exposure.

The main study limitations were the relatively small population and that it was not possible to recruit more participants with a high-sugar intake. This means that the results might only be relevant for average sugar consumers, whereas data for high-sugar consumption are still lacking. It was surprisingly difficult to recruit high-sugar consumers to the study, and most subjects screened were of mid-level socioeconomic status with a healthy life-style and low DMFT scores. There are well-documented limitations with the use of self-reported dietary questionnaires, and underreporting the intake of sugars and fat is often inversely related to the participants’ body weight [32,33]. To address the known limitations of questionnaires, a validated quantitative food frequency questionnaire with 180 food items was used, and the calculations of nutrients and energy intake were performed with aid of software (DANKOST) based on the Danish food composition tables. The sugar intake data are therefore considered reasonably robust and reliable. Nevertheless, further research involving subjects with very high and frequent consumption of sugars would be helpful to improve the understanding on the impact of refined sugars on the composition of the oral microbiota.

For the bacterial identification in the plaque samples, NGS combined with species-specific sequences using the PROBESEQ program for identifications (HOMI*NGS*) was used [17]. This technique was recently developed, combining advances of DNA reads, generated through NGS (Illumina platform), and a subsequent BLAST of generated 16S rRNA reads against reference sequences of species-specific, custom-designed 16S rDNA ‘probes’, enabling simultaneous identification of approximately 600 oral taxa at the species level. Previous studies utilizing HOMI*NGS* have shown that the salivary microbiota is host specific and without major diurnal variations [34]. Furthermore, the qPCR analyses for detection of selected taxa have proven highly useful for mapping bacterial biomarkers in early childhood caries [15,22,^26^,^35^]. With this technique, *S. wiggsiae* has previously been linked to advanced [22] and initial caries [36], and thus the present findings extend these data to a reference group compared to a low caries-conducive diet.

## Conclusions

The amount of ingested sugars may influence the microbial profile in dental plaque and saliva from young adults. In subjects with low intake of free sugars, caries-associated species with acidogenic and aciduric properties were less common than among those with a more excessive intake. The findings support the concept of caries as a biofilm-mediated disease, driven by environmental stress of the commensal oral bacteria.

## Supplementary Material

Supplementary_files.zip
