# Supplementary material for: Oral microbial profiles of individuals with different levels of sugar intake
Source: J Oral Microbiol. 2017 Aug 1;9(1):1355207. doi: 10.1080/20002297.2017.1355207 (PMC5560414; doi:10.1080/20002297.2017.1355207)
Supplement: Supplementary_files.zip [file zjom_a_1355207_sm5116.zip › Supplementary files/Supplementary file 1.pdf]

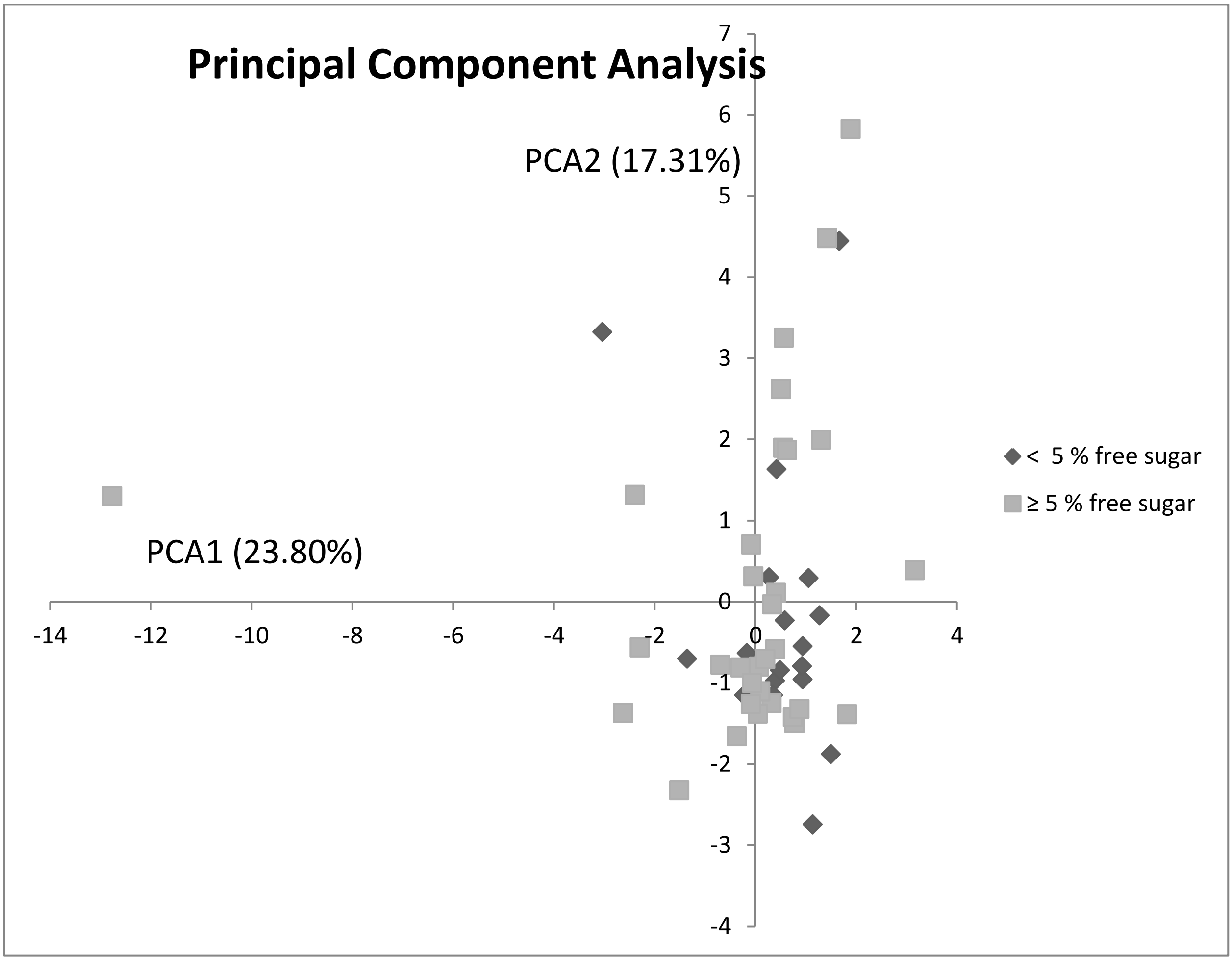

Principal component analysis visualized two-dimensionally with axes expressed as the two foremost components accounting for 41.11% of the variation of the dataset. Samples from the low sugar group (dark grey) and samples from the reference sugar group samples (light grey).
