# Supplementary material for: Oral microbial profiles of individuals with different levels of sugar intake
Source: J Oral Microbiol. 2017 Aug 1;9(1):1355207. doi: 10.1080/20002297.2017.1355207 (PMC5560414; doi:10.1080/20002297.2017.1355207)
Supplement: Supplementary_files.zip [file zjom_a_1355207_sm5116.zip › Supplementary files/Supplementary file 2.pdf]

28-items FFQ used for screening volunteers. Translated from Danish.

**How often do you eat any of the following food items?**								
How often?<br>Food items	Never	1 time or less per month	2 times per month	1 time per week	2-3 times per week	1 times per day	2-3 times per day	4 times or more per day
Butter								
Cheese								
Milk								
Yoghurt								
Rye bread (plain)								
Rye bread (whole grain)								
Toast								
Potatoes								
Vegetables								
Fruit								
Dried fruit								
Rice								
Spaghetti								
Beef								
Fish								
Ice cream								
Cake								
Cookies								
Pastries								
Marmalade, honey								
Soft drink (sugar free)								
Soft drink								
Fruit syrup (sugar free)								
Fruit syrup								
Juice								
Chocolate								
Candy								
Sugar in coffee/tea								

1. Have you changed your diet dramatically within the last 2-3 years?
   1 □ yes2 □ no
2. How many meals do you eat per day?
   ____
3. How often do you snack between meals?

   ____
